# Analysis of Gut Microbiome Using Explainable Machine Learning Predicts Risk of Diarrhea Associated With Tyrosine Kinase Inhibitor Neratinib: A Pilot Study

**DOI:** 10.3389/fonc.2021.604584

**Published:** 2021-03-10

**Authors:** Chi Wah Wong, Susan E. Yost, Jin Sun Lee, John D. Gillece, Megan Folkerts, Lauren Reining, Sarah K. Highlander, Zahra Eftekhari, Joanne Mortimer, Yuan Yuan

**Affiliations:** ^1^ Department of Applied AI and Data Science, City of Hope National Medical Center, Duarte, CA, United States; ^2^ Department of Medical Oncology & Therapeutic Research, City of Hope National Medical Center, Duarte, CA, United States; ^3^ Pathogen and Microbiome Division, Translational Genomics Research Institute North, Flagstaff, AZ, United States

**Keywords:** gut microbiota, breast cancer, neratinib, diarrhea, artificial intelligence, explainable machine learning

## Abstract

Neratinib has great efficacy in treating HER2+ breast cancer but is associated with significant gastrointestinal toxicity. The objective of this pilot study was to understand the association of gut microbiome and neratinib-induced diarrhea. Twenty-five patients (age ≥ 60) were enrolled in a phase II trial evaluating safety and tolerability of neratinib in older adults with HER2+ breast cancer (NCT02673398). Fifty stool samples were collected from 11 patients at baseline and during treatment. 16S rRNA analysis was performed and relative abundance data were generated. Shannon’s diversity was calculated to examine gut microbiome dysbiosis. An explainable tree-based approach was utilized to classify patients who might experience neratinib-related diarrhea (grade ≥ 1) based on pre-treatment baseline microbial relative abundance data. The hold-out Area Under Receiver Operating Characteristic and Area Under Precision-Recall Curves of the model were 0.88 and 0.95, respectively. Model explanations showed that patients with a larger relative abundance of *Ruminiclostridium* 9 and *Bacteroides* sp. HPS0048 may have reduced risk of neratinib-related diarrhea and was confirmed by Kruskal-Wallis test (p ≤ 0.05, uncorrected). Our machine learning model identified microbiota associated with reduced risk of neratinib-induced diarrhea and the result from this pilot study will be further verified in a larger study.

**Clinical Trial Registration:**

ClinicalTrials.gov, identifier NCT02673398.

## Introduction

Neratinib is a potent small molecule tyrosine kinase inhibitor (TKI) that inhibits human epidermal growth factor receptors (HER1, HER2, and HER4). Neratinib has recently been granted FDA approval as extended therapy for early stage HER2+ breast cancer and in combination with capecitabine for treatment of HER2+ metastatic breast cancer (1–3). Despite excellent efficacy data, neratinib is associated with significant gastrointestinal (GI) toxicity, with grades 1–4 diarrhea observed in 95% of patients and grade 3–4 diarrhea in over 40% of patients in earlier trials (3–5). In nine trials of neratinib alone or in combination with other therapy, dose reductions due to diarrhea ranged from 20%–53% (1, 3, 6–12). Analysis of the ExteNET trial showed neratinib-associated diarrhea had a distinct and predictable clinical course, with 28.6% of patients having grade 3 events during the first month, then decreasing to ≤ 6% after month 3. Grade 3 events are generally short-lived and occur within the first month of treatment, allowing targeted preventive management with antidiarrheal prophylaxis early in the treatment course (13).

Older adults with breast cancer undergoing therapy with neratinib are particularly vulnerable to severe diarrhea due to potential changes in absorption, drug metabolism and distribution with increased age. An investigator-initiated clinical trial was designed to evaluate the safety and tolerability of neratinib in adults 60 and older with metastatic HER2+ breast cancer (NCT02673398). A total of 25 patients were enrolled and here we report gut microbiome analysis using 16S rRNA gene sequencing of longitudinally collected stool specimen in 11 patients.

The human gut contains a dense microbiome ecosystem that is essential in maintaining a healthy host physiology, and disruption of this ecosystem has been linked with increased risk of toxicities from systemic cancer therapy (14–19). The bacteriomic profile of the gut microbiome can be an indicator of general health and disease such as inflammation, digestive inefficiencies, and the presence of pathogens. The advent of next-generation sequencing technologies such as 16S rRNA gene or metagenome sequencing have enabled characterization of the gut microbiome architecture in an affordable and culture-free approach (20).

The objective of this study was to understand the association of host gut microbiome and neratinib-induced diarrhea. Utilizing 16S rRNA gene sequencing data, a machine learning model was built for prediction of diarrhea from baseline intestinal microbiota data in breast cancer patients.

## Materials and Methods

### Patients

A phase II, single arm, open label study was conducted between 09/2015 and 12/2019 (NCT02673398). Eligibility criteria were histologically proven metastatic breast cancer; HER2+ defined by ASCO/CAP guideline; age ≥ 60; Eastern Cooperative Oncology Group (ECOG) performance status (PS) 0–2. Patients were started with a neratinib dose of 240 mg oral daily in a 28-day cycle. Diarrhea prophylaxis with loperamide was mandatory during the first cycle of treatment and was used as needed beyond the first cycle. Adverse events (AEs) were assessed by NCI Common Terminology Criteria for Adverse Events (CTCAE) 4.0. This study was approved by City of Hope’s regulatory and ethics committees and was conducted in accordance with the Declaration of Helsinki and the principles of Good Clinical Practice. All participants provided written informed consent.

### Sample Collection

Stool samples were collected at baseline, Cycle 1 Day 15, Cycle 2 Day 1, Cycle 2 Day 15, and Cycle 3 Day 1. Fifty samples from 11 patients were collected using Zymo DNA/RNA Shield Fecal Collection tubes. For the machine learning model, stool samples and patient adverse event of diarrhea at any time over the course of treatment were analyzed.

### Microbiome Analysis

DNA was extracted using the MagMax Microbiome Ultra Extraction Kit (AA2358, Thermo-Fisher, Waltham, MA), with prior bead beating on a TissueLyser (Qiagen), using the KingFisher Magnetic Extraction Instrument (ThermoFisher). DNAs were quantitated by the BactQuant assay (21). 16S rRNA libraries were created as described by Kozich et al. (22), using modified primers described by Walters et al. (23), that amplify the 16s rRNA variable region 4 (V4) of the rRNA gene of bacteria and archaea. More than 16,000 reads per sample were produced. Reads were quality filtered, trimmed and chimeras removed then taxonomically classified using the Silva database using QIIME2 (24, 25). The relative abundance of 26 taxa present at ≥ 5% in any one sample within this cohort was reported to the genus level or above. Shannon’s diversity index was also calculated to examine gut microbiome dysbiosis.

### Explainable Tree-Based Predictive Modeling

To classify which patients would have neratinib-related diarrhea, microbial relative abundance data from the 11 patients (50 specimens) were utilized for predictive modeling. Input features were fed into machine learning models to classify which patients would have diarrhea. A non-parametric and non-linear gradient-boosted tree approach [xgboost package (26)] was utilized for classification. For model performance assessment, a leave-one-patient-out approach was chosen. In each iteration, input data from one patient was held out while data from the rest of the patients were used for training a model. Default set of regularization and hyperparameters were used to fit the model. The fitted model was then applied only on the pre-treatment baseline data from the hold-out patient. Receiver Operating Characteristic (ROC) and Precision-Recall Curves (PRC) were used for model assessment. Subsequently, the entire dataset was used to fit a final model. A tree-explainer was used to compute local explanations [probabilistic SHAP value, shap package (27, 28)] based on the associated exact shapley values generated for each feature from individual patient’s data. The overall feature importance was obtained by calculating the mean absolute SHAP values of individual features. Post-hoc analyses were then performed on the most important features using Kruskal-Wallis tests to assess the differences of the most important microbiota relative abundance between patients without and without neratinib-induced diarrhea. Python 3.7.6, scikit-learn 0.22.1, xgboost 1.0.1, shap 0.35.0, and scipy 1.4.1 were used for machine learning modeling and statistical analysis.

## Results

### Patients

A total of 11/25 patients who were accrued between December 2016 and March 2019 provided 50 longitudinal stool samples. Patient and disease characteristics are summarized in **Supplementary Table 1**. The median age was 66 years old (60–78). Sixty-four percent were Caucasian and 36% were Asian, with 27% of Hispanic ethnicity. Eighty-two percent were hormone receptor positive HER2+ metastatic breast cancer. Seventy-three percent developed grade ≥ 1 diarrhea attributed to neratinib over the course of treatment.

### Microbiome Analysis

**Figure 1:**
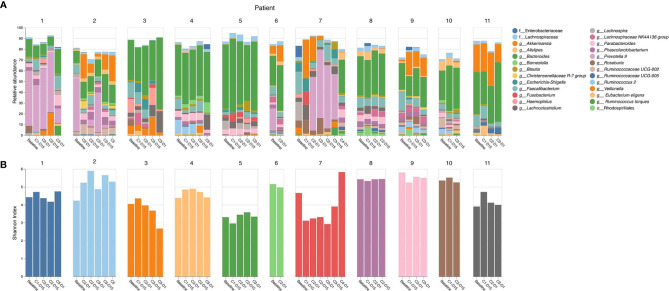
16S rRNA gene sequencing analysis. **(A)** Relative abundance of top 26 taxa by patient and cycle; **(B)** Shannon’s alpha diversity by patient and cycle.

16S rRNA gene analysis was performed and the relative abundance of 26 taxa present at ≥ 5% in any one sample within this cohort was reported to the genus level or higher as classified by QIIME2 (25) (**Figure 1A**). *Bacteroides* (green) is a common anaerobic gut inhabitant that comprises a large proportion of the human gut microbiome, is responsible for fermentation of long chain carbohydrates, produces butyrate, and dominates in populations who eat a Western diet. *Bacteroides* sp. were present in most patients in this study (9/11, 82%), and were predominant at baseline and throughout treatment. *Prevotella* 9 (pink/violet) is a member of the same family as *Bacteroides*, but is more prevalent in non-Westernized populations. Two out of 11 (18%) patients had *Prevotella* during treatment. *Akkermansia* (orange) is associated with a healthy gut, and has anti-inflammatory effects. Five out of 11 (45%) patients had *Akkermansia* during treatment.

Shannon’s alpha diversity (29) measures the evenness in the fecal communities. Shannon diversity was calculated to examine gut microbiome dysbiosis (**Figure 1B**). A normal healthy gut microbiome usually has a Shannon diversity index of 3.5 or greater. Several samples fell below this threshold; however, no clear association of Shannon alpha diversity index and diarrhea was identified.

### Explainable Prediction Model for Diarrhea Due to Neratinib

**Figure 2:**
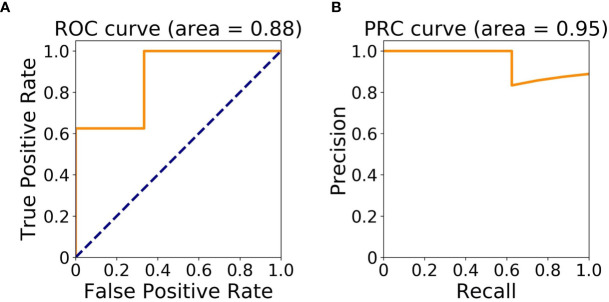
Model assessment. **(A)** Area Under Receiver Operating Characteristic (ROC) Curve; **(B)** Area Under Precision-Recall Curve (PRC).

The cohort for machine learning modeling included patients 1, 2, 5, 7–11 with at least one occurrence of diarrhea (n = 8), and patients 3, 4, 6 with no diarrhea (n = 3). The Area Under ROC (AUROC) and Area Under PRC (AUPRC) of the classification model were 0.88 and 0.95, respectively **(** **Figures 2A, B**).

**Figure 3:**
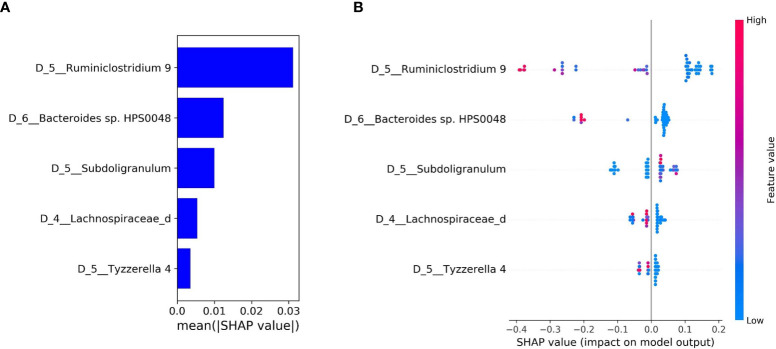
Feature importance and local explanation of final model. **(A)** Bar plot of mean absolute SHAP values of individual features; and **(B)** Beeswarm plot showing feature values and impact on the model prediction.

**Figure 4:**
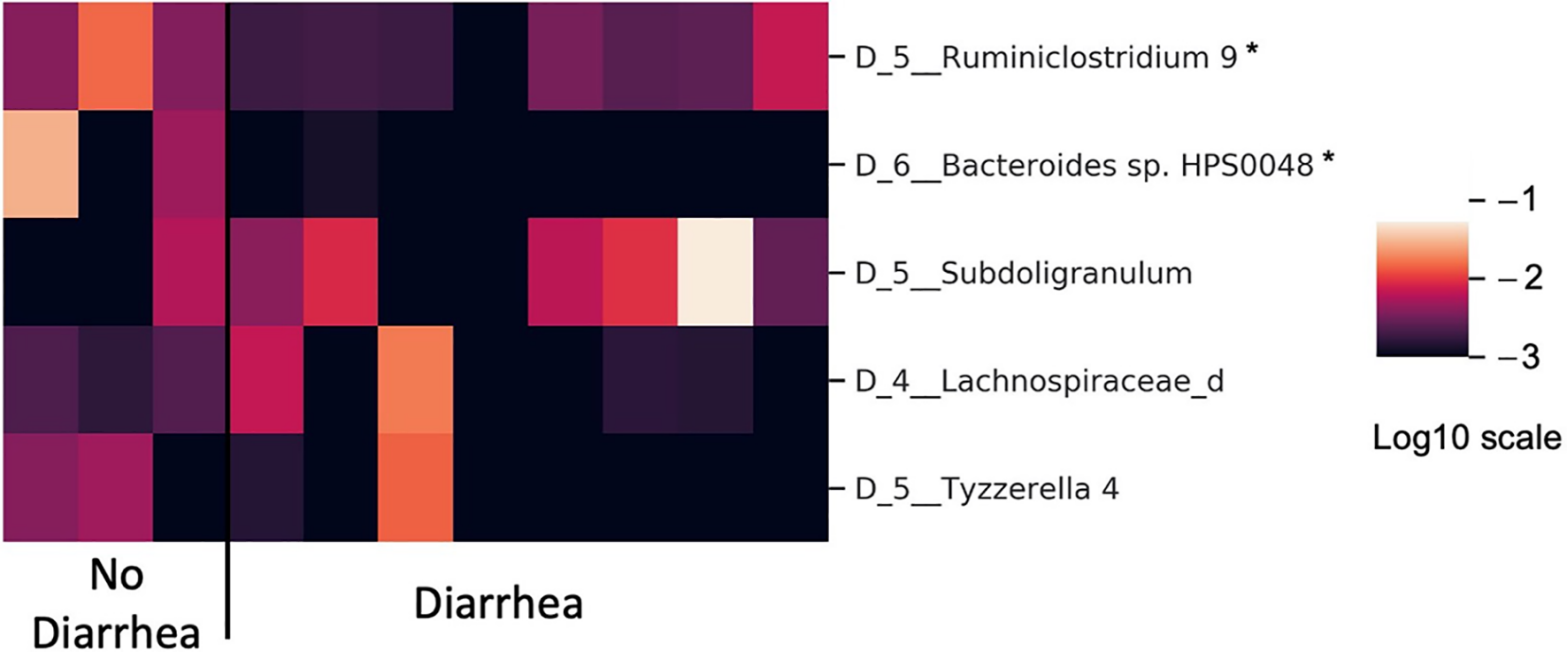
Heatmap showing differences in microbiota relative abundance between patients with and without neratinib-induced diarrhea (in log10 scale). *Kruskal-Wallis test with p ≤ 0.05 uncorrected.

The bar plot of mean absolute SHAP values of individual features associated with the final optimal model suggests *Ruminiclostridium* 9 and *Bacteroides* sp. HPS0048 are the two most impactful features overall for predicting risk for treatment-related diarrhea (**Figure 3A**). A beeswarm plot (27) shows each row corresponding to one feature and each dot corresponding to one patient in the full dataset (**Figure 3B**). The color of each dot corresponds to normalized feature value (qualitative: blue for low values, red for high values) whereas its position along the x-axis depicts the SHAP value describing the impact on the model prediction. Using the beeswarm plot, the directionality of the relation between individual features and outcome can be observed, along with the magnitude of the relation. Specifically, patients with larger relative abundance of *Ruminiclostridium* 9 and *Bacteroides* sp. HPS0048 may have reduced risk of treatment-related diarrhea. A heatmap displays the relative abundance of *Ruminiclostridium* 9 and *Bacteroides* sp. HPS0048 in log10 scale (**Figure 4**). Using Kruskal-Wallis tests, statistically significant differences between patients with and without diarrhea for *Ruminiclostridium* 9 (p = 0.04, uncorrected) and *Bacteroides* sp. HPS0048 (p = 0.05, uncorrected) were identified. No statistically significant difference was observed for the next three important features (D_5__Subdoligranulum: p = 0.21; D_4__Lachnospiraceae_d: p = 0.21, D_5__Tyzzerella 4: p = 0.24, uncorrected).

## Discussion

Using the relative abundance of microbiome taxa data, we have developed an explainable tree-based predictive model to estimate the risk of diarrhea associated with neratinib treatment in patients ≥ 60 with HER2+ metastatic breast cancer. Utilizing a nested cross-validation approach, we achieved a promising 0.88 AUROC and 0.95 AUPRC for model performance. We also identified the most important features that could predict diarrhea and the risk with the associated directionality: patients with higher relative abundance of *Ruminiclostridium* 9 and *Bacteroides* sp. HPS0048 may have a reduced risk of treatment-related diarrhea. The findings were confirmed by Kruskal-Wallis tests (p ≤ 0.05, uncorrected). Both species produce short chain fatty acids (SCFAs) including acetate, propionate, and butyrate, which are important metabolites for maintaining intestinal homeostasis. In addition, butyrate also has important immunomodulatory functions and may activate signaling cascades that control immune functions (30).

Multiple studies have shown that the gut microbiota has the potential to influence the efficacy of cancer therapy (31, 32). There are several potential mechanisms of action for TKI induced diarrhea, including direct target inhibition, chloride secretion, intestinal inflammation, and a dysbiotic microbiome (31). Both preclinical and clinical studies have demonstrated decreased total bacterial abundance and diversity, as well as decreases in commensals such as *Lactobacillus* and Bifidobacteria, with increases in Bacteroidetes and *Escherichia coli* contributing to TKI-induced diarrhea (32). The translocation, immunomodulation, metabolism, enzymatic degradation, reduced diversity (TIMER) model proposed by Alexander and colleagues has outlined how the functions of the microbiome may have a central role in determining the extent and intensity of diarrhea induced by systemic therapy (33). Heshiki et al. investigated the contribution of the intestinal microbiome on treatment outcomes in a heterogeneous cohort that included multiple cancer types to identify microbes with a global impact on immune response (34). This human gut metagenomic analysis revealed that responders had significantly higher microbial diversity and different microbiota compositions compared to non-responders, and species such as *Bacteroides ovatus* and *Bacteroides xylanisolvens* were positively correlated with treatment outcomes. In the current study, no clear association of Shannon alpha diversity index and diarrhea was identified. This likely reflects the relatively small sample size.

Machine learning and deep learning models are being widely used for precision oncology research (35). Although the predictive models often have impressive predictive performance, they are typically difficult to explain. Explaining predictive models is one of the key factors driving their use in a clinical setting (36, 37). To correlate model prediction accuracy and explainability, various approaches have been proposed to generate intuitive interpretations on predictive models (27, 28, 38, 39). Using a concept in game theory, Lungberg et al. proposed a unified framework called SHAP (SHapley Additive exPlanations) that works for essentially all predictive models including tree-based and deep learning models (38). In SHAP, each feature is assigned an importance value (SHAP value) and the addition of all SHAP values leads to the actual prediction. A model prediction can be decomposed into a unique set of SHAP values associated with the features, allowing clinicians and scientists to utilize contributions of individual feature SHAP values and their interactions to draw insight from the dataset.

The current pilot study is limited by its small sample size. Longitudinal samples were collected from 11 patients with 50 specimens. To reduce potential bias due to training a predictive model with limited sample size, we have adopted a robust train-test split approach where the test dataset in each leave-one-patient-out loop was kept entirely independent of the train dataset (40). Increased cohort size and an external dataset would boost the utility of this model. Nevertheless, the high model performance (AUROC and AUPRC) with meaningful feature explanation offers a foundation for future studies. Our study also contributes to the new field of gut microbiome in oncology by providing a novel predictive model associating host gut microbiota with treatment-induced diarrhea. TKIs have been broadly used in current oncology practice and diarrhea is a common and challenging side effect. Modulation of the microbiota may support cancer therapy and improve patient’s quality of life in the future (34).

## Conclusion

Our machine learning model identified microbiota associated with reduced risk of neratinib-induced diarrhea and the result from this pilot study will be further verified in a larger study.

## Data Availability Statement

The original contributions presented in the study are publicly available. This data can be found here: https://github.com/cwwong-alec/Analysis-of-Gut-Microbiome-Using-xML-Predicts-Risk-of-Diarrhea-Associated-With-TKI-Neratinib

## Ethics Statement

The studies involving human participants were reviewed and approved by City of Hope’s IRB-approved protocol. All procedures were carried out in accordance with The Code of Ethics of the World Medical Association (Declaration of Helsinki) and Recommendations for the Conduct, Reporting, Editing, and Publication of Scholarly Work in Medical Journals. Privacy rights were observed and written informed consent was obtained from all participants of this study under IRB15342 and ClinicalTrials.gov NCT02673398. The patients/participants provided their written informed consent to participate in this study.

## Author Contributions

CW and YY made substantial contributions to conception and design, analysis, and interpretation of data, drafting and revising the manuscript, and final approval for publication. JL and SY contributed to database management, chart review, data analysis, manuscript preparation, and revision. JG, MF, LR, and SH performed the 16S rRNA gene sequencing and analysis. ZE, JM, and SH revised the manuscript and provided final approval for publication. YY is the guarantor and agrees to be accountable for all aspects of the work. All authors contributed to the article and approved the submitted version.

## Funding

Puma sponsored the trial and provided neratinib. This study was supported by COH Pathology Research Services Core and Biostatistics and Mathematical Modeling Core (National Cancer Institute of the National Institutes of Health under award number P30CA033572). The authors also thank the NIH R03 AG050931-02(PI YY) STOP Cancer Foundation (PI YY), NIH K-12 Career Development Award (K12CA001727, PI JM), and NIH 1R01CA206911-01A1 (PI Emily Wang). Puma was not involved in the study design, collection, analysis, interpretation of data, the writing of this article or the decision to submit it for publication.

## Conflict of Interest

YY has contracted research sponsored by Merck, Eisai, Novartis, Puma, Genentech, Celgene, and Pfizer; is a consultant for Puma, Pfizer, Immunomedics, and is on the Speakers Bureau for Eisai, Novartis, Genentech, AstraZeneca, Daiichi Sankyo, Pfizer, Merck and Immunomedics.

The remaining authors declare that the research was conducted in the absence of any commercial or financial relationships that could be construed as a potential conflict of interest.
