# Supplementary material for: Analysis of Gut Microbiome Using Explainable Machine Learning Predicts Risk of Diarrhea Associated With Tyrosine Kinase Inhibitor Neratinib: A Pilot Study
Source: Front Oncol. 2021 Mar 10;11:604584. doi: 10.3389/fonc.2021.604584 (PMC8008168; doi:10.3389/fonc.2021.604584)
Supplement: Supplementary file 1 [file DataSheet_1.docx]

**Supplemental Table 1: Patient characteristics**

| Characteristic | Patients (n = 11) |
| --- | --- |
| Median age (range) | 66 (60-78) |
| Gender  Female | 11 (100%) |
| Race  Caucasian  Asian | 7 (64%)  4 (36%) |
| Ethnicity  Non-Hispanic  Hispanic | 8 (73%)  3 (27%) |
| Hormone receptor status  Positive  Negative | 9 (82%)  2 (18%) |
| BMI status  Normal  Overweight  Obese | 5 (46%)  4 (36%)  2 (18%) |
| Adverse event > grade 1  Diarrhea  No diarrhea | 8 (73%)  3 (27%) |
